# Magnetic Nanoparticles in Agriculture: Unraveling the Impact of Nickel Ferrite Nanoparticles on Peanut Growth and Seed Nutritional Quality

**DOI:** 10.3390/plants14071011

**Published:** 2025-03-24

**Authors:** Yuying Tang, Taiming Zhang, Yuanbo Li, Quanlong Wang, Weichen Zhao, Muhammed Nadeem, Peng Zhang, Yukui Rui

**Affiliations:** 1 Beijing Key Laboratory of Farmland Soil Pollution Prevention and Remediation, College of Resources and Environmental Sciences, China Agricultural University, Beijing 100193, China; tangyuying@cau.edu.cn (Y.T.); liyuanbo@cau.edu.cn (Y.L.);; 2 State Key Laboratory for Environmental Chemistry and Ecotoxicology, Research Center for Eco-Environmental Sciences, Chinese Academy of Sciences, Beijing 100085, China; 3 School of Geography, Earth and Environmental Sciences, University of Birmingham, Edgbaston, Birmingham B15 2TT, UK; 4 Department of Environmental Science and Engineering, University of Science and Technology of China, Hefei 230026, China

**Keywords:** magnetic nanoparticles, peanut, seed nutritional quality, plant growth regulation, sustainable agriculture

## Abstract

Nanotechnology has been a source of innovation in various fields in recent years, and its application in agriculture has attracted much attention, particularly for its potential to enhance crop growth and optimize nutritional quality. This study systematically investigated the effects of nickel ferrite nanoparticles (NiFe_2_O_4_ NPs) on peanut (*Arachis hypogaea* L.) growth, nutrient dynamics, and biochemical responses, highlighting their potential as sustainable alternatives to conventional fertilizers. The results showed that an optimum concentration of 50 mg/kg soil significantly improved photosynthetic efficiency, biomass accumulation, seed yield, and nutritional quality, with 1000 seed weight and total yield increasing by 12.3% and 15.6%, respectively. In addition, we hypothesized that NiFe_2_O_4_ NPs would activate the antioxidant system and increase plant resistance. According to the risk assessment, the target hazard quotient (THQ = 0.081) is well below the safety threshold of 1. These findings provide strong evidence for the application of NiFe_2_O_4_ NPs as next-generation nano-fertilizers, offering a dual advantage of improved agronomic performance and biosafety. However, further research is needed to optimize their application strategies and assess potential long-term environmental impacts.

## 1. Introduction

Global population growth and escalating climate change present significant challenges to agricultural sustainability. Among these, iron deficiency in soils is a critical constraint, negatively impacting plant growth, reducing yields, and lowering iron bioavailability in edible portions—thereby exacerbating iron-deficiency anemia, a major global public health issue [1,2,3]. Current iron supplementation strategies rely heavily on inorganic salts, chelated complexes, and organic formulations, with chelates such as ferric ethylenediaminetetraacetic acid (EDTA-Fe) and ethylenediamine-N,N’-bis(2-hydroxyphenylacetic acid) iron (EDDHA-Fe) demonstrating superior stability and bioavailability [4,5,6]. However, their widespread adoption is hindered by high production costs, limited efficiency in calcareous soils, and environmental concerns associated with the persistence of synthetic chelators [7,8]. As global agriculture shifts toward sustainable intensification, there is a growing need for cost-effective, environmentally friendly iron delivery systems.

Nanotechnology has emerged as a transformative approach in agronomy, with iron-based nanomaterials—such as nano-Fe_3_O_4_ and γ-Fe_2_O_3_—demonstrating remarkable potential as next-generation nano-fertilizers [9,10,11,12]. Among these, NiFe_2_O_4_ NPs have gained increasing attention due to their unique physicochemical properties, including superparamagnetism, high chemical stability, and nanoscale-controlled reactivity [13,14,15,16]. In plant systems, iron and nickel are critical micronutrients; iron is indispensable for chlorophyll biosynthesis, electron transport, and enzymatic activation in photosynthesis [17,18,19], while nickel serves as a cofactor for urease activity and a regulator of stress-responsive pathways [20,21,22]. Harnessing the dual functions of NiFe_2_O_4_ NPs may offer a synergistic approach to micronutrient delivery and oxidative stress mitigation, ultimately enhancing crop growth, resilience, and yield stability.

Beyond agriculture, NiFe_2_O_4_ NPs have been extensively studied in biomedical applications, particularly in targeted drug delivery and nanomedicine [23,24,25,26,27] These nanoparticles have demonstrated multifunctional capabilities, including pH-responsive drug release for cancer therapy and reactive oxygen species (ROS) modulation [28], as well as precision pulmonary delivery of dexamethasone for respiratory infections, achieving >80% alveolar deposition efficiency in murine models [29]. Importantly, comprehensive toxicological evaluations have consistently reported negligible cytotoxicity (*p* > 0.05 vs. controls), with cellular viability exceeding 95% at therapeutic concentrations. This well-documented biocompatibility provides a crucial safety justification for their transition from biomedical to agricultural applications.

Peanut (*Arachis hypogaea* L.) is a globally important cash crop that is utilized across a variety of fields, including agricultural production, the food industry, medicine, and cosmetics. However, the application of nanomaterials in major food crops (e.g., rice, wheat, and maize) has been the subject of extensive research, while research on peanuts is comparatively limited. The existing studies have predominantly focused on nano-oxides and nano-mono materials [30,31,32], with research on the application of magnetic nanoparticles in agriculture being in its infancy. The exploration of the effect of NiFe_2_O_4_ NPs as a novel iron nano-fertilizer on peanut yield and quality is, therefore, a significant scientific undertaking with considerable application potential.

The present study was thus conducted on peanuts, with the aim of exploring the potential of NiFe_2_O_4_ NPs as a nano-fertilizer and providing new ideas for the efficient production and quality improvement of peanuts. The study will systematically evaluate the effects of NiFe_2_O_4_ NPs on the growth and development of peanuts and further resolve the possible physiological and biochemical regulatory mechanisms. The objective of this study was to evaluate the effects of different concentrations of NiFe_2_O_4_ NPs (0, 5, 50, and 200 mg/kg) on peanut growth and development. At the conclusion of the complete growth cycle of the peanut, its phenotypic characteristics and physiological indices were systematically analyzed. The results of this study will contribute to a deeper understanding of the effects of NiFe_2_O_4_ NPs on peanut growth and provide a scientific basis for their application in agriculture.

## 2. Result and Discussion

### 2.1. Nanoparticle Characterization

**Figure 1:**
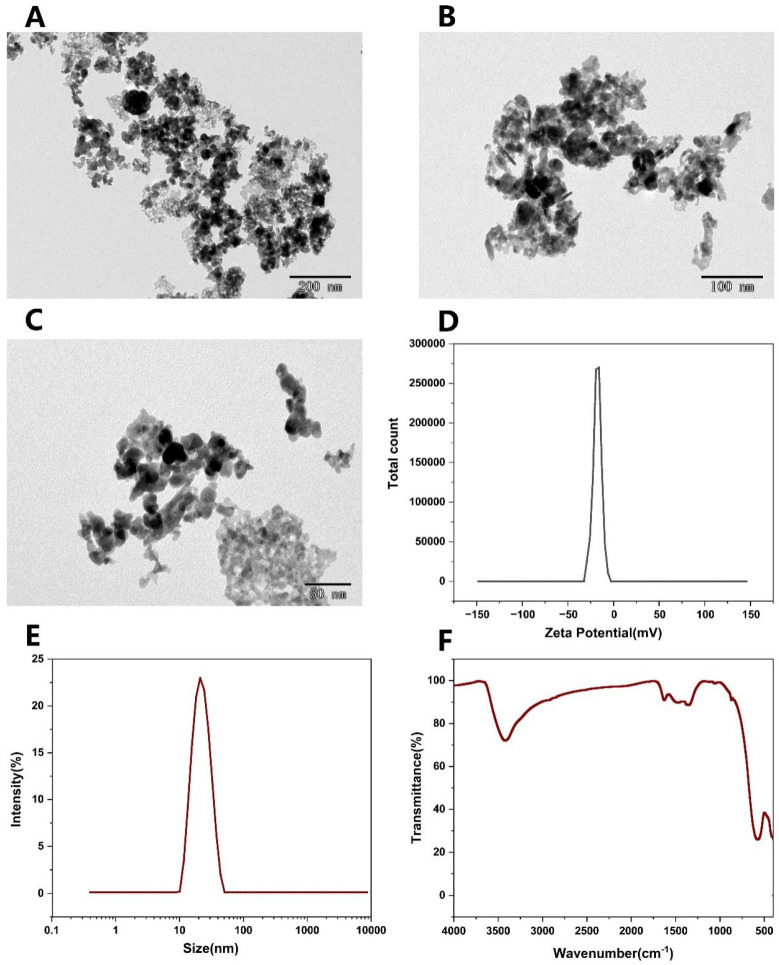
TEM electron micrograph of NiFe_2_O_4_ NPs. (**A**) Scale bar  =  200 nm. (**B**) Scale bar = 100 nm, (**C**) Scale bar = 50 nm. Zeta potential (**D**), hydrodynamic diameter (**E**), and FTIR spectrometry (**F**) of NiFe_2_O_4_ NPs [33].

A comprehensive physicochemical analysis of NiFe_2_O_4_ nanoparticles (NPs) elucidated their structure, which is critical for their potential agricultural applications (Figure 1). Transmission electron microscopy (TEM) (Figure 1A–C) confirmed a well-defined cubic spinel structure, with primary particle sizes ranging from 20 to 50 nm. Moderate aggregation was observed, likely due to their inherently high surface energy.

Colloidal stability assessments further characterized their dispersion behavior, with a ζ-potential of −16.5 ± 4.68 mV, indicating moderate electrostatic stabilization (Figure 1D). The particle size was 20.09 nm, with a low polydispersity index (PDI = 0.059), as determined by dynamic light scattering (DLS) (Figure 1E), suggesting a uniform size distribution. Fourier-transform infrared spectroscopy (FTIR) revealed characteristic peaks at 3400 cm^−1^ (broad O–H stretching band), which are indicative of surface hydroxylation (Figure 1F). This suggests that surface hydroxylation may facilitate hydration-mediated stabilization and influence nanoparticle interactions in aqueous environments.

These well-defined physicochemical properties, including nanoscale dimensions, controlled agglomeration, and surface functionality, suggest that NiFe_2_O_4_ NPs could be effectively leveraged in agricultural systems, offering optimized nutrient delivery and enhanced environmental compatibility.

### 2.2. Growth Modulation

**Figure 2:**
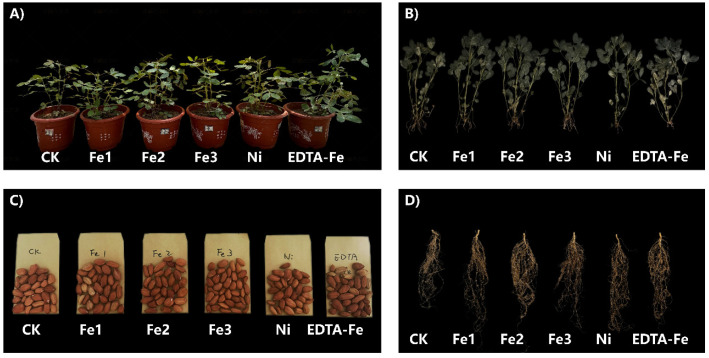
Phenotypic images of peanuts treated with different concentrations of nano iron–nickel ferrite materials for 120 days. Fe1/Fe2/Fe3 (5/50/200 mg/kg NiFe_2_O_4_ NPs), Ni (33 mg/kg NiSO_4_), and EDTA-Fe (156 mg/kg EDTA-Fe). (**A**), pots. (**B**), shoots. (**C**), seeds. (**D**), roots.

To evaluate the impact of NiFe_2_O_4_ NPs on peanut growth, this study systematically assessed the morphological and biomass responses of both aerial and subterranean plant components under different treatment conditions. Phenotypic characterization based on smartphone-captured digital images (Figure 2) revealed that plants treated with Fe1, Fe2, and Fe3 exhibited enhanced vigor compared to the control (CK) plants, which were characterized by lush foliage, robust root development, and well-formed seeds.

**Figure 3:**
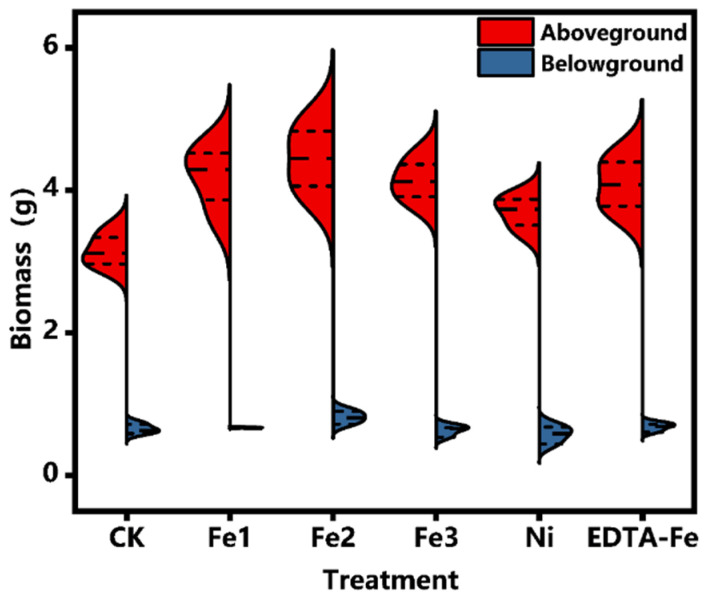
The effects of the various treatments on the dry weight of the aboveground part and the dry weight of the underground part.

Biomass quantification (Figure 3) further corroborated these observations, demonstrating a significant increase in shoot dry weight for the Fe1–Fe3 treatments, indicating a dose-dependent growth-promoting effect of NiFe_2_O_4_ NPs. Notably, EDTA-Fe treatment resulted in comparable shoot biomass to that in the Fe-treated groups, reinforcing iron’s essential role in enhancing plant productivity. These findings underscore the importance of optimizing NiFe_2_O_4_ NP application rates, with Fe2 emerging as the most effective treatment. The concentration-dependent response patterns highlight the necessity for precise dosage control in agricultural applications of engineered nanomaterials.

### 2.3. Effects of NiFe_2_O_4_ NPs on Peanut Photosynthetic Pigments and Gas Exchange Parameters

Chlorophyll content (SPAD values) and gas exchange parameters are critical indicators of photosynthetic capacity and overall physiological status in plants [34,35,36,37]. This study systematically assessed SPAD values at 30, 60, and 90 days post-treatment, alongside the net photosynthetic rate (Pn) and stomatal conductance (Gs) at 120 days, to elucidate the dose-dependent effects of NiFe_2_O_4_ NPs on peanut photosynthesis.

**Figure 4:**
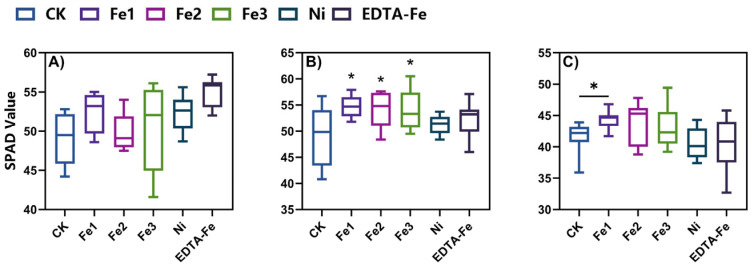
Relative chlorophyll contents (SPAD values) at 30 (**A**), 60 (**B**), and 90 (**C**) days post-treatment. Asterisks (*) indicate statistically significant differences compared to the control group: * *p* < 0.05. The number of asterisks corresponds to the level of significance.

At 30 days (Figure 4A), Fe3-treated plants exhibited a 2.8% increase in SPAD values compared to CK plants, while EDTA-Fe achieved the highest chlorophyll content, suggesting superior iron bioavailability in chelated form during early growth stages. Nickel treatment induced only marginal enhancement (vs. CK treatment), indicating its limited role in chlorophyll biosynthesis at this stage. By day 60 (Figure 4B), Fe3’s advantage expanded to 10.2% over CK values, whereas EDTA-Fe’s effect plateaued, likely due to rapid nutrient depletion. Fe2 treatment led to a 10.7% increase from baseline, suggesting time-dependent activation of nanoparticle-mediated iron release. At the reproductive stage (day 90, Figure 4C), CK plants exhibited natural chlorophyll degradation due to senescence, whereas Fe2- and Fe3-treated plants maintained 5.2% and 2.9% higher SPAD values than CK plants, confirming NiFe_2_O_4_ NPs’ role in delaying senescence. EDTA-Fe’s equivalence to CK treatment reinforced its transient efficacy, while Ni-treated plants exhibited accelerated chlorophyll loss, indicating chronic phytotoxicity.

**Figure 5:**
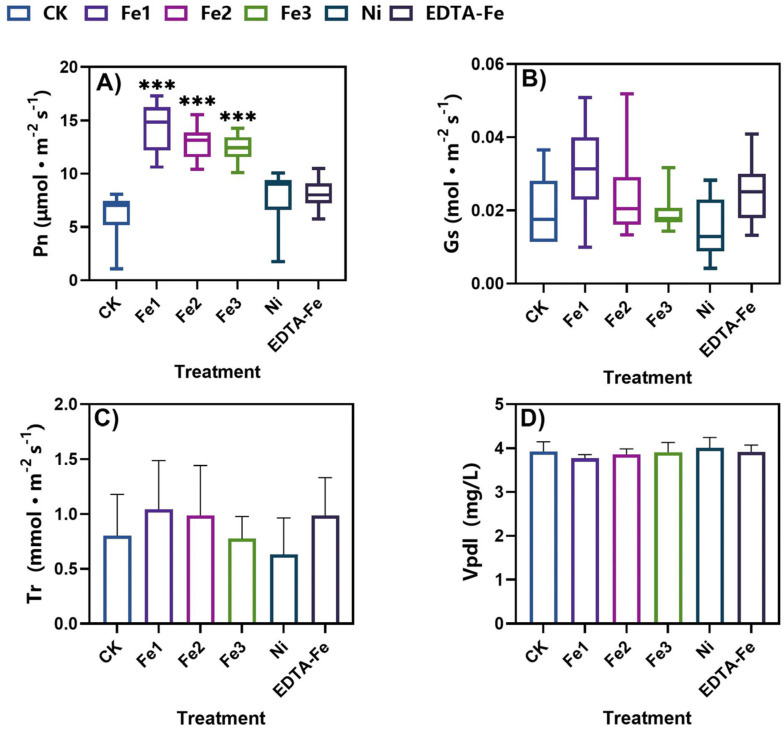
Effects of NiFe_2_O_4_ NPs on the net photosynthetic rate (**A**), stomatal conductance (**B**), the transpiration rate (**C**), and the water vapor pressure difference (**D**) in peanut leaves. Asterisks (*) indicate statistically significant differences compared to the control group: *** *p* < 0.001. The number of asterisks corresponds to the level of significance.

At harvest (120 days, Figure 5), the 5 mg/kg, 50 mg/kg, and 200 mg/kg treatments significantly increased the plant net photosynthetic rate (Pn) by 132%, 107%, and 100%, respectively, compared to CK treatment, which may be due to optimized chlorophyll synthesis and stomatal regulation. In addition, the 5 mg/kg treatment significantly increased Gs by 70.1%, Fe2 treatment maintained values comparable to those with CK treatment, with a difference of only 1.6%, and the 200 mg/kg treatment reduced Gs by 26.3%, suggesting that high concentrations of nanoparticles may induce stomatal closure. The 5 mg/kg and 50 mg/kg treatments also maintained a normal transpiration rate (Tr) and leaf vapor pressure deficit (Vpdl). These changes indicated that 50 mg/kg NiFe_2_O_4_ NPs effectively improved photosynthetic efficiency by promoting chlorophyll retention while reducing stomatal disturbance. The sustained release properties of the nanoparticles provided a more stable supply of iron than EDTA-Fe, which provided only transient benefits. These concentration-dependent responses highlight the need for precise dose optimization in nano-agriculture.

### 2.4. Effects of NiFe_2_O_4_ NPs on Peanut Yield

**Figure 6:**
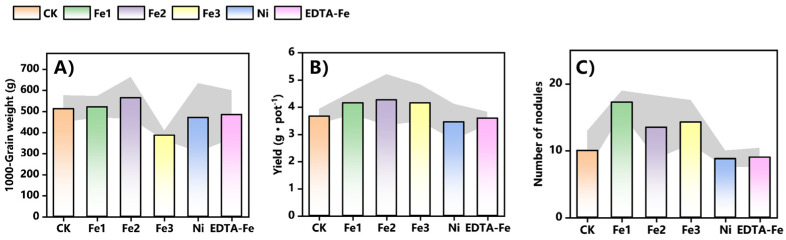
Effects of NiFe_2_O_4_ NPs on the peanut antioxidant enzyme system. (**A**), Shoot SOD activity. (**B**), Root SOD activity. (**C**), Shoot POD activity. (**D**), Root POD activity. (**E**), Shoot CAT activity. (**F**), Root CAT activity. (**G**), Shoot MDA content. (**H**), Root MDA content. Asterisks (*) indicate statistically significant differences compared to the control group: * *p* < 0.05, ** *p* < 0.01, *** *p* < 0.001, **** *p* < 0.0001. The number of asterisks corresponds to the level of significance. Effects of NiFe_2_O_4_ NPs on 1000 seed weight (**A**), yield (**B**), and nodule number (**C**) after full-life cycle cultivation.

Based on the yield data in Figure 6A,B, both Fe1 and Fe2 treatments had a positive effect on groundnut yield. Compared to the control (CK), Fe1 treatment increased thousand kernel weight, yield, and the number of rhizomes by 1.7%, 13%, and 72.5%, respectively, whereas Fe2 treatment resulted in 5.1%, 16%, and 35% increases, respectively. Notably, Fe1 treatment caused greater enhancement of rhizome formation than Fe2 treatment, suggesting a stronger effect on nitrogen fixation potential, which could contribute to nutrient uptake efficiency and yield improvement. On the other hand, Fe2 treatment showed more pronounced effects on yield and kernel weight, likely due to the optimized iron release kinetics of NiFe_2_O_4_ NPs, ensuring a continuous and efficient iron supply for plant growth.

However, at high concentrations (Fe3 treatment), a negative impact on yield was observed, indicating that excessive nanoparticle application may exert detrimental effects. This highlights the importance of dosage optimization to maximize benefits while minimizing potential toxicity.

Overall, the NiFe_2_O_4_ NPs demonstrated significant potential in enhancing peanut yield, likely through a synergistic mechanism involving improved nutrient regulation and root system development.

### 2.5. Effects of NiFe_2_O_4_ NPs on the Peanut Antioxidant System

Plants are constantly exposed to environmental stressors that trigger excessive accumulation of reactive oxygen species (ROS), such as hydroxyl radicals (·OH) and superoxide anions (O_2_^−^) [38]. ROS can disrupt cellular structures, damage lipids, proteins, and nucleic acids, and ultimately impair plant growth or induce programmed cell death (PCD) [39,40,41,42,43,44,45]. Plants have developed a very effective antioxidant defense system that includes both enzymatic and non-enzymatic components to combat oxidative stress [46,47,48]. Superoxide dismutase (SOD), catalase (CAT), and peroxidase (POD) are key antioxidant enzymes that facilitate ROS detoxification, while non-enzymatic antioxidants contribute to cellular redox homeostasis [49,50].

**Figure 7:**
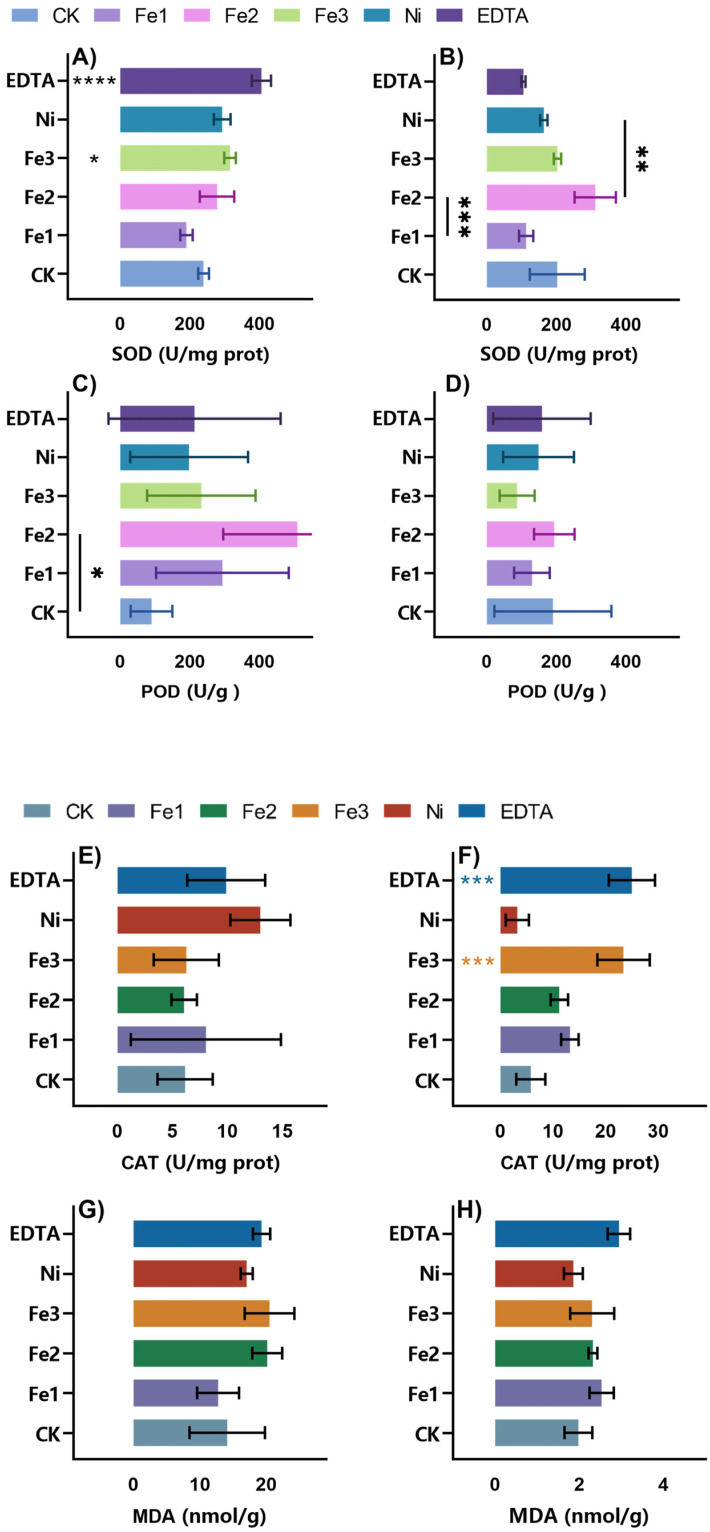
Effects of NiFe_2_O_4_ NPs on the peanut antioxidant enzyme system. (**A**), Shoot SOD activity. (**B**), Root SOD activity. (**C**), Shoot POD activity. (**D**), Root POD activity. (**E**), Shoot CAT activity. (**F**), Root CAT activity. (**G**), Shoot MDA content. (**H**), Root MDA content. **** *p* < 0.0001 *** *p* < 0.001 ** *p* < 0.01 * *p* < 0.05.

This study investigated the concentration-dependent effects of NiFe_2_O_4_ NPs on the peanut antioxidant system (Figure 7). Malondialdehyde (MDA) accumulation, an indicator of lipid peroxidation and oxidative stress, exhibited a dose-dependent pattern, while Fe1 and Fe2 treatments resulted in MDA levels comparable to or slightly lower than those with control (CK) treatment, and Fe3 treatment significantly increased MDA accumulation, suggesting that excessive nanoparticle exposure exacerbates membrane oxidative damage.

Antioxidant enzyme activity measurements further underscored the dual role of NiFe_2_O_4_ NPs in modulating oxidative stress. Compared to that in CK plants, CAT activity was significantly elevated in Fe1-treated plants, while SOD activity reached its highest levels under Fe2 treatment, indicating an enhanced ROS scavenging capacity at moderate nanoparticle concentrations. Additionally, POD activity followed a similar upward trend under Fe1 and Fe2 treatments but declined sharply in the Fe3 group, reinforcing the notion that high nanoparticle concentrations may impair enzymatic defenses.

In summary, NiFe_2_O_4_ NPs exerted a concentration-dependent regulatory effect on the peanut antioxidant system. Low-to-moderate concentrations (Fe1, Fe2) bolster antioxidant defenses by upregulating SOD, CAT, and POD activity, whereas high concentrations (Fe3) induce oxidative damage, as evidenced by increased lipid peroxidation and enzymatic inhibition. These findings provide critical insights into the physiological impact of nanomaterials in agriculture, highlighting the necessity of precise dosage control to harness their benefits while mitigating potential phytotoxicity.

### 2.6. Effects of NiFe_2_O_4_ NPs on Mineral Nutrient Elements in Peanut Grains

Mineral nutrients are essential to the growth of plants and physiological metabolism. This study employed inductively coupled plasma mass spectrometry (ICP-MS) to analyze mineral element contents in peanut tissues (leaves, stems, roots, and grains) in order to evaluate the effects of NiFe_2_O_4_ NPs on the absorption and distribution of mineral nutrients in peanuts.

**Figure 8:**
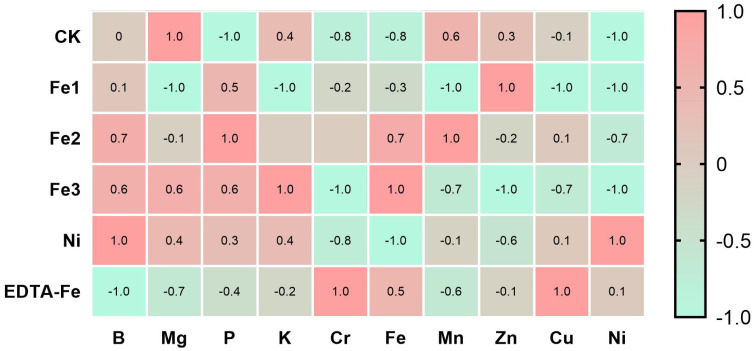
Distribution of the standardized contents of major mineral elements in peanut seeds under different treatments.

For a more intuitive analysis of the effects of NiFe_2_O_4_ nanoparticles (NPs) on mineral nutrient elements in peanut kernels, the data in this study were standardized using the normalization method (range −1 to 1) to compare the relative trends of element contents in different treatment groups. As shown in Figure 8.

Overall, the Ni treatment group (Ni) showed the highest accumulation of Ni elements with a normalized value close to 1.0, as well as enhanced uptake of B elements and also increased accumulation of K and P, while the control group (CK) had the lowest contents. In addition, the Fe2- and Fe3-treated groups showed different increases in B, P, K, and Fe contents compared to the CK group, indicating that the supply of NiFe_2_O_4_ NPs had a certain promoting effect on the accumulation of these elements, although the accumulation effects were different depending on the concentration applied. In addition, EDTA-Fe treatment also had a certain enhancing effect on the accumulation of Ni, Cr, and Cu, which may be related to the fact that EDTA as a chelating agent can enhance the mobility of heavy metals and cause changes in their distribution in plant tissues.

In conclusion, different nano metallic materials and chelating treatments exerted complex effects on the distribution of mineral elements in peanut kernels; Ni treatment significantly increased the accumulation of Ni and some nutrients, Fe treatment showed different effects in terms of Fe, Mn, and Zn, and EDTA-Fe treatment promoted the uptake of some heavy metals. Future studies could further explore the potential effects of these changes on the nutritional quality and food safety of peanuts.

### 2.7. Effects of NiFe_2_O_4_ NPs on Peanut Seed Organic Nutrient and Amino Acid Contents

**Figure 9:**
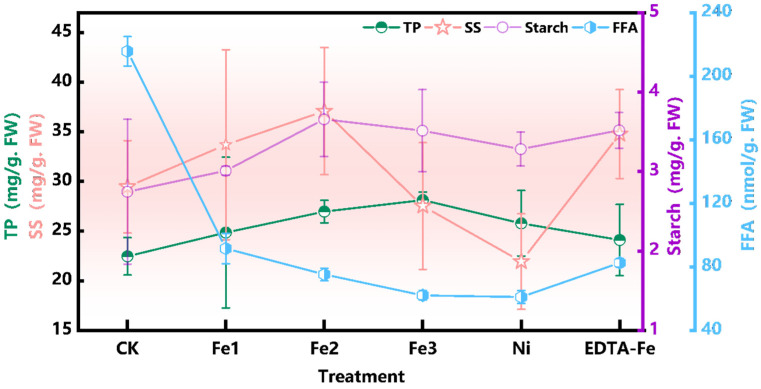
Effects of NiFe_2_O_4_ NPs on starch content, total protein concentration, soluble sugar content, and free fatty acids in seeds after full-life cycle cultivation.

This study examined the effects of NiFe_2_O_4_ NPs on the macronutrient composition of peanut seeds, focusing on total protein (TP), soluble sugar (SS), starch (Starch), and free fatty acids (FFAs), as shown in Figure 9. These components are key indicators of nutritional quality and storage stability, making their evaluation essential for assessing the agricultural potential of NiFe_2_O_4_ NPs.

Protein content is one of the important indicators for measuring peanut nutritional value [51,52,53]. Fe2 treatment led to a notable increase in the total protein content by approximately 20%, suggesting that optimal NP concentrations possibly increase the number of rhizomes, which in turn increases nitrogen uptake, thus promoting protein biosynthesis. Fe3 treatment further increased the TP content by 25.2%, indicating that higher NP concentrations may continue to promote protein accumulation. Fe1 and Ni treatments induced moderate increases, while EDTA-Fe treatment had a relatively minor effect.

Soluble sugar is one of the important metabolic products affecting peanut flavor and energy storage [54,55]. Fe1, Fe2, and EDTA-Fe treatments increased soluble sugar levels by approximately 10.8%, 19.6%, and 13.6%, respectively. However, Fe3 treatment resulted in a 5% decrease in the SS content compared to CK treatment, suggesting that excessive NP concentrations may interfere with sugar metabolism.

Starch content, a key determinant of peanut processing quality [56,57,58], was highest in Fe2-treated seeds, with a 33.6% increase over CK values. Fe3 treatment also significantly increased the starch content by 28.1%, while Fe1 treatment resulted in a smaller increase of 9.7%. Other treatments led to smaller, statistically nonsignificant increases. This suggests that NiFe_2_O_4_ NPs may enhance overall carbohydrate metabolism, improving both nutritional and industrial value.

Higher FFA levels accelerate lipid oxidation, reducing storage stability [59,60,61]. Fe3 treatment significantly lowered the FFA content by 71.2% compared to CK treatment, while Fe1, Fe2, and EDTA-Fe treatments also showed notable reductions (57.6%, 65.1%, and 62.8%, respectively). These findings suggest that NiFe_2_O_4_ NPs may mitigate lipid degradation, possibly by inhibiting lipid-degrading enzymes.

Moderate NiFe_2_O_4_ NP levels (50 mg/kg, Fe2) enhanced TP, SS, and starch accumulation while decreasing the FFA content, effectively reducing lipid degradation, and improving storage stability. Higher concentrations (Fe3) may disrupt nutrient accumulation. These results highlight the potential of NiFe_2_O_4_ NPs in optimizing crop quality. However, further research is needed to elucidate their molecular mechanisms and assess long-term ecological impacts for safe and sustainable agricultural applications.

### 2.8. Peanut Seed Risk Assessment

The mean nickel contents of peanut kernels in different treatment groups were 1.41 mg/kg (CK), 1.49 mg/kg (Fe1), 1.95 mg/kg (Fe2), 1.47 mg/kg (Fe3), 4.94 mg/kg (Ni), and 4.26 mg/kg (EDTA-Fe). For an adult weighing 60 kg who consumes 50 g (0.05 kg) of peanuts per day, the intake of nickel would be 1.95 mg/kg:

$$EDI=\frac{1.945\times 0.05}{60}=0.00162\;mg/kg/cdotpday\;THQ=\frac{0.00162}{0.02}=0.081<1$$

The result is less than 1, indicating a low health risk. Consequently, the administration of NiFe_2_O_4_ NPs does not pose a threat to human health.

In conclusion, it is evident that the ingestion of peanut seeds may present certain health risks should the levels of contaminants in peanut seeds exceed the established safety thresholds. Consequently, a systematic risk assessment is imperative to ensure the safety of peanuts and to provide a scientific foundation for risk management.

## 3. Conclusions

This study explored the regulatory effects of NiFe_2_O_4_ NPs on peanut growth, physiological characteristics, and seed nutritional quality. The results showed that at an appropriate concentration (50 mg/kg), NiFe_2_O_4_ NPs could effectively replace traditional iron fertilizers, significantly improving the photosynthetic efficiency, yield, and key nutritional components of peanut seeds. In addition, they enhanced stress resistance by activating antioxidant enzymes such as SOD, CAT, and POD. However, excessive concentrations led to oxidative stress, suggesting a dose-dependent effect that needs careful consideration.

Risk assessment indicated that the nickel accumulation in peanut seeds (THQ = 0.081) remained well below safety thresholds, implying that the potential health risk is low under controlled conditions. These findings suggest that NiFe_2_O_4_ NPs could serve as an alternative iron supplement in agriculture, offering a promising approach to improving both crop productivity and nutritional value.

Despite these positive outcomes, future research needs to further fully ascertain the underlying mechanisms of NiFe_2_O_4_ NPs in plant growth regulation. The application of omics techniques could help reveal their broader effects on plant resistance to drought and salinity. Moreover, their interactions with rhizosphere microbial communities should be investigated to assess potential ecological impacts.

From a practical perspective, optimizing application methods, understanding their release dynamics and degradation patterns, and evaluating potential synergies with other biostimulants are necessary steps to ensure safe and efficient use. Long-term environmental impact assessments should also be conducted, including impacts on soil health, risks of bioaccumulation, and potential impacts on the food chain, as well as their intergenerational effects and enrichment factors. Additionally, large-scale field trials under different environmental conditions are essential to validate these findings and provide guidance for real-world agricultural applications.

In conclusion, NiFe_2_O_4_ NPs show great potential as a nano-fertilizer for improving crop yield and nutritional quality. However, their long-term ecological effects and practical feasibility must be further explored to ensure safe and sustainable agricultural applications. Future research should focus on bridging the gap between experimental studies and field applications, paving the way for the responsible use of nanotechnology in modern agriculture.

## 4. Materials and Methods

### 4.1. Characterization of Nanoparticles

The NiFe_2_O_4_ NPs used in this study (purity ≥ 99.99%, average particle size: 20 nm) were purchased from Bide Pharmatech Ltd. (Shanghai, China) and synthesized via the co-precipitation method. All other chemicals were of analytical grade. The morphology and primary particle size of the nanoparticles were analyzed using transmission electron microscopy (JEM-1200EX, JEOL, Tokyo, Japan). The hydrated particle size and surface potential were determined using a Zetasizer Nano ZS90 particle size analyzer (Malvern, UK) with 100 mg/L deionized water as the dispersing medium. Chemical functional groups were identified through Fourier-transform infrared spectroscopy (VERTEX 70, Boston, MA, USA).

### 4.2. Experimental Design

A pot experiment was conducted under controlled greenhouse conditions at the West Campus of China Agricultural University (CAU), Beijing. Peanut seeds (*Arachis hypogaea* L. cv. ‘Huayu 25’) were obtained from Shandong Native Specialty Store (via Pinduoduo) and underwent the following pretreatment:

- Surface sterilization in 10% (*v*/*v*) H_2_O_2_ for 10 min, followed by eight rinses with deionized water;
- Hydration in darkness at 25 ± 1 °C for 12 h;
- Germination in Petri dishes (100 mm × 15 mm) lined with moist filter paper [62] and incubation at 25 ± 1 °C (DRP-9052 incubator, Peiyin, China) until radicle elongation reached 1 cm.

The growth medium consisted of soil (collected from CAU Shangzhuang Experimental Station: 40°13’38.9″ N, 116°17’86.6″ E) mixed with quartz sand (Pipi Organic Substrate, Taobao) at a 1:5.5 (*w*/*w*) ratio [11,63]. The soil characteristics are shown in Table 1. Basal fertilization was applied at a rate of N: P_2_O_5_:K_2_O = 0.25:0.3:0.25 mg/kg [38] using urea [9], superphosphate, and potassium sulfate. The experiment included the following six treatments with 2 kg of substrate per pot (230 mm × 180 mm): Control (CK) (no supererogatory Fe/Ni supplementation), Fe1/Fe2/Fe3 (5/50/200 mg/kg NiFe_2_O_4_ NPs), Ni (33 mg/kg NiSO_4_), and EDTA-Fe (156 mg/kg EDTA-Fe).

After a 14-day soil equilibration period (greenhouse conditions: 25/18 °C day/night, 60% RH), four pre-germinated seeds were sown per pot. At 38 days after sowing (DAS), seedlings were thinned to one per pot [64]. The experiment followed a completely randomized design with four biological replicates per treatment.

### 4.3. Chlorophyll Quantification

Chlorophyll content (SPAD values) was measured at three growth stages (30, 60, and 90 DAS) using a SPAD-502 Plus chlorophyll meter (Konica Minolta, Tokyo, Japan). Measurements were taken from the third fully expanded trifoliate leaf from the apex, with two readings per leaflet and three biological replicates per treatment. Data collection followed manufacturer protocols under consistent ambient light (1000–1200 μmol⋅m^−2^⋅s^−1^ PAR) [65].

### 4.4. Gas Exchange Analysis

At 90 DAS, photosynthetic parameters (net photosynthetic rate [Pn], stomatal conductance [Gs], and intercellular CO_2_ concentration [Ci]) were measured using the LI-6400XT portable photosynthesis system (LI-COR Biosciences, USA) [66,67]. Measurements were conducted between 09:00 and 11:00 AM under saturating light (1500 μmol⋅m^−2^⋅s^−1^) and a controlled CO_2_ concentration (400 ppm). To correlate chlorophyll contents with photosynthetic efficiency, ten SPAD readings were averaged per leaf.

### 4.5. Yield Component Analysis

Peanut samples were collected at the following two time points [68,69]:

1. 45 DAS: Leaves were sampled for chlorophyll measurement and then stored at −20 °C for physiological and biochemical analyses;
2. 120 DAS (full life cycle): Plants were washed with deionized water, and surface soil was removed. Roots were further cleaned with 0.1% dilute nitric acid to eliminate adsorbed ions and nanoparticles. Plant height, root length, and grain yield were recorded. Tissues were dried at 105 °C for 1 h and then at 70 °C for 24 h before determining the dry weight.

### 4.6. Antioxidant Enzyme Profiling

Fresh tissue (0.1 g) was homogenized in ice-cold PBS (1:10 *w*/*v*, pH 7.8) using a ball mill (MM400, Retsch, Haan, Germany). After centrifugation (8000× *g*, 10 min, 4 °C), supernatants were analyzed for malondialdehyde (MDA) levels and the enzyme activities of superoxide dismutase (SOD), peroxidase (POD), and catalase (CAT) [38,70,71,72,73] using commercial kits (Comin Biotechnology, Suzhou, China). Absorbance was measured with a U-2910 microplate reader (Hitachi, Chiyoda, Japan).

### 4.7. Elemental Composition Analysis

The elemental composition of peanut plants and seeds was analyzed following the procedure by Zhou et al. [69]. Plant samples were initially dried at 105 °C for 30 min, followed by a second drying at 75 °C for an additional 30 min until a stable weight was achieved. Approximately 0.2 g of the dried material was placed into a digestion tube, and 8 mL of high-purity concentrated nitric acid was added. The tube was sealed with a notched stopper and left in the dark overnight. The following day, the material was further decomposed using a microwave digestion system (MARS6, CEM, Buckingham, UK); the samples were initially heated at 120 °C for 30 min, followed by an increase to 140 °C, which was maintained for 3 h, and finally, the acid was evaporated at 170 °C until the solution volume was reduced to 1 mL. The solution was then diluted to a final volume of 50 mL with ultrapure water. After filtration through a 0.25-micron polytetrafluoroethylene membrane, it was diluted again with ultrapure water. The concentrations of nickel, iron, and other minerals were measured using an Elan DRC-e inductively coupled plasma mass spectrometer (ICP-MS) from Perkin Elmer, Waltham, MA, USA [33,74].

### 4.8. Nutritional Metabolite Assays

The protein content in peanut seeds was measured using the BCA assay. Under alkaline conditions, certain amino acids and peptide bonds in proteins reduce Cu^2+^ ions to Cu^+^, which then form a purple complex with bicinchoninic acid (BCA) [75,76]. Specifically, 0.1 mL of bovine serum albumin (BSA) solution (0.01 mg/mL) was mixed with 2.0 mL of a solution containing reagent A (1% BCA) and reagent B (4% CuSO_4_) in a 50:1 ratio. Approximately 0.125 g of tissue was homogenized in 1 mL of distilled water on ice, the sample was centrifuged at 10,000 rpm for 10 min at 4 °C, and the supernatant was collected. After incubation with BSA at 60 °C for 30 min, the absorbance was measured at 562 nm using a microplate reader (EPOCH-SN, BioTek, Winooski, VT, USA) [77,78].

Soluble sugar and starch contents in peanut seeds were determined as follows [79]. Approximately 0.125 g of peanut seeds was homogenized in 1 mL of distilled water, heated in a water bath at 95 °C for 10 min, and centrifuged at 8000× *g* for 10 min at 25 °C [80]. The supernatant was diluted and used to measure the soluble sugar content by adding a color-developing agent and heating it in a water bath at 95 °C for 10 min. The absorbance was measured at 620 nm. For the starch content, the residue was gelatinized in water at 100 °C and treated with perchloric acid, and the absorbance was measured at 620 nm after adding anthrone reagent and sulfuric acid [81].

### 4.9. Risk Assessment of Peanut Seeds

Nanomaterials can enter plants via roots, leaves, or soil–plant systems and accumulate in different organs. Iron (Fe) and nickel (Ni) uptake and translocation in peanut tissues were analyzed to evaluate potential risks associated with NiFe_2_O_4_ NP exposure [82,83].

The health risk of chemical pollutants is assessed using the estimated daily intake (EDI):

(1) $$EDI=\left(C\times IR\right)/BW$$

where C is the pollutant concentration in seeds (mg/kg), IR is the daily intake (kg/day), and BW is body weight (kg).

Non-carcinogenic risk is evaluated using the target hazard quotient (THQ):

(2) THQ=EDI/RfD

The RfD (reference dose) is the reference dose (mg/kg-day) that is provided by the relevant authorities (the United States Environmental Protection Agency (EPA) has set the RfD for nickel at 0.02 mg/kg-day); if THQ ≥ 1, a health risk may exist.

### 4.10. Statistical Analysis

The experimental results are presented as the mean ± standard deviation, with four replicates for each treatment. Statistical analysis was conducted using SPSS 27.0 software. Differences between groups were evaluated by one-way analysis of variance (ANOVA) and Tukey’s test. In the figures, asterisks (*) indicate statistically significant differences (*p* < 0.05) between the treatment groups and the control group.

## Figures and Tables

**Table 1:** Soil characteristics.

Indicator	Sand (Average Value)	Soil (Average Value)
Soil type	Fine sand	Sandy chernozem
pH	1.68	8.38
Total organic matter (g/kg)	26.6	8.36
Available potassium (AK) (mg/kg)	1.06	66.07
Available phosphorus (AP) (mg/kg)	1.68	11.76

## Data Availability

The datasets presented in this article are not easily made public due to confidentiality agreements. Requests to access the datasets should be directed to the first author.

## Abbreviations

The following abbreviations are used in this manuscript:

NPs	Nanoparticles
NiFe_2_O_4_ NPs	Iron–nickel oxide nanoparticles
Tris-HCl	Tris (hydroxymethyl) aminomethane hydrochloride
DLS	Dynamic light scattering
CAT	Catalase
MDA	Malondialdehyde
SOD	Superoxide dismutase
POD	Peroxidase
SS	Soluble sugar
TP	Total protein
Starch	Starch
AA	Amino acid
FFA	Free fatty acid
AK	Available potassium
AP	Available phosphorus
